# Sparganosis (*Spirometra* spp.) in Asian Water Monitor (*Varanus salvator*): A medical implications for veterinarians, breeders, and consumers

**DOI:** 10.14202/vetworld.2021.2482-2487

**Published:** 2021-09-23

**Authors:** Aditya Yudhana, Ratih Novita Praja, Anjani Marisa Kartikasari

**Affiliations:** 1 Department of Veterinary Science, Division of Veterinary Parasitology, Faculty of Veterinary Medicine, Universitas Airlangga, Surabaya, Indonesia; 2 Department of Veterinary Science, Division of Veterinary Microbiology, Faculty of Veterinary Medicine, Universitas Airlangga, Surabaya, Indonesia; 3 Department of Veterinary Science, Faculty of Veterinary Medicine, Universitas Airlangga, Kampus C Mulyorejo Street, Surabaya, East Java, Indonesia.

**Keywords:** infectious disease, sparganosis, *Spirometra*, *Varanus salvator*

## Abstract

**Background and Aim:**

The high prevalence of sparganosis has been reported globally, especially in Asian countries where the majority of individuals consume raw meat from wild-caught reptiles. In Indonesia, similar cases regarding the high prevalence of sparganosis were recorded from wild reptiles such as snakes that utilized for culinary purposes, whereas, there are no data regarding other species such as water monitor lizard (*Varanus salvator*), which also provided as daily culinary with a high number of trades. Therefore, this study aims to investigate the prevalence of sparganosis in water monitor lizard (*V. salvator*), which is frequently utilized as culinary products in East Java Province, Indonesia.

**Materials and Methods:**

A total of 313 living wild-caught and captive-bred Asian water monitor lizards were collected from the reptile markets and breeders. All samples were euthanized and observed for the presence of plerocercoid. Identification of the plerocercoid as larval infective stage was made using carmine staining method.

**Results:**

The total prevalence of sparganosis was recorded at 69.64%. A total of 393 plerocercoids were collected in which divided 280 (71.24%) infecting muscles and 113 (28.75%) located in subcutaneous tissues.

**Conclusion:**

To the best of our knowledge, this study not only recorded as the first evidence but also confirms the role of monitor lizards as sparganosis transmitters in Asia and reveals additional routes of sparganosis transmission in Indonesian reptiles. Local conservation laws should be strengthened to effectively control or ban wildlife trade in traditional markets. Moreover, public awareness regarding sparganosis as a neglected zoonotic disease should be applied to prevent disease transmission in Indonesia.

## Introduction

Tapeworm of the genus of *Spirometra* has been recognized as intestinal parasites not only in domestic mammals (i.e., dogs and cats) [1] but also in wildlife (i.e., reptiles and amphibians), with a worldwide distribution [2]. The plerocercoid larvae, commonly known as spargana, correspond to the infective stage and can infect humans, causing sparganosis [3]. Although the majority of human cases are categorized as rare, sparganosis remains endemic in many countries, particularly in Southeast Asia [4-6]. *Spirometra* life cycle requires two different intermediate hosts under the natural condition: The first intermediate host is freshwater copepods, and amphibians, reptiles, birds, and mammals play a role as the second intermediate host [7,8]. Humans, as the paratenic host for *Spirometra*, can be infected by the parasite in three ways. The first possibility is the ingestion of infected wild animal products such as raw snakes and frogs that contain spargana; second, infected copepods are ingested from contaminated drinking water; and third, by applying flesh or skin of infected frogs to a wound or eyesores as traditional medicine, which may increase the risk of spargana migration to various visceral organs, including subcutaneous tissues, causing nodules or swellings. Moreover, based on the complexity of the parasite life cycle, sparganosis also categorized as food- and water-borne zoonotic disease [9,10].

A high prevalence of sparganosis has been previously reported in Guangdong Province, China, where the majority of local people consume raw meat from wild-caught snakes. Moreover, reptile meats in several Asian countries also regarded as popular traditional culinary foods that have a delicious taste, becoming the main reason for local restaurants providing wild-caught snakes or other reptiles as their primary menu [11-13]. Many people enjoy eating half-cooked or even completely raw meat, skin, and gallbladder of wild-caught reptiles, without considering the high risk of infection by *Spirometra* parasites [14]. Reptiles and amphibians, as intermediate hosts for *Spirometra* tapeworm, are already considered important sources for sparganosis transmission, due to their zoonotic potential [15]. In Indonesia, similar cases regarding the high prevalence of sparganosis have been recorded from local wild animals, particularly snakes, such as *Ptyas mucosus*, *Dendrelaphis pictus*, and *Naja sputatrix*, contributing a total prevalence of 68%, 50.85%, and 56.7%, respectively [16-18]. Sparganosis cases were also recorded from Asian wild frogs (*Rana rugulosa*) meats which sold in several local restaurants in East Java Province with a 9.1% prevalence rate [19,20]. Interestingly, *P. mucosus* and *N. sputatrix* are considered the most common wild-caught snakes that are utilized as public culinary in Indonesia, whereas other species of reptiles, such as *Varanus salvator* are commonly known as water monitor lizard, are also provided as daily culinary items with extensive trading.

Therefore, the present study aimed to investigate the occurrence of sparganosis in *V. salvator*, which is generally sold and utilized for culinary purposes. To the best of our knowledge, there have been no scientific studies regarding sparganosis in water monitor lizards from Indonesia. Thus, the results of the present study may contribute to the identification of the sources of sparganosis infection, which also provides important implications for strengthening proper programs regarding the prevention of sparganosis, which is a neglected zoonotic disease in East Java Province, Indonesia.

## Materials and Methods

### Ethical approval

This study was conducted with permission from the local agriculture and wildlife departments in East Java Province, Indonesia. This study was reviewed and approved by the Animal Care and Use Committee of the Faculty of Veterinary Medicine, Universitas Airlangga, Indonesia (No.1.KE.006.01.2020).

### Study location and sampling

A total of 313 living wild-caught and captive-bred Asian water monitor lizards were collected from reptile markets and breeders in East Java Province, Indonesia. The specific sampling locations were selected based on the biggest reptile markets selling water monitor lizards at Mojokerto City (112.434084 longitude and −7.472638 latitude), Sidoarjo City (112.667542 longitude and −7.472613 latitude), Gresik City (112.655472 longitude and −7.156576 latitude), and Banyuwangi City (114.369227 longitude and −8.219233 latitude). Asian water monitor lizard (*V. salvator*) species were identified according to their morphological characteristics, particularly the color patterns on the dorsal and ventral sides. The back and the dorsal side of the limbs have light spots and blotches between the forelimbs and hindlimbs. The head and neck have dark transverse bands on the snout and temporal stripes behind the eyes. The color pattern of the chin generally has light-dark V-shaped markings at the lower jaw that may build cross bands. The throat is mostly light with dark markings of small dots, bigger blotches, V-shaped elements, or cross bands [21].

### Plerocercoid collection

**Figure-1:**
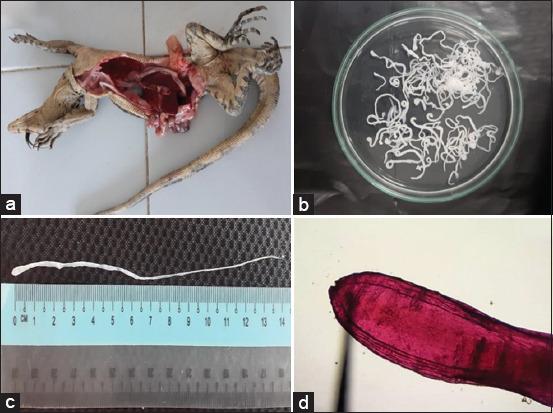
(a) *Varanus salvator* sample during necropsy, (b) plerocercoids collected from *Varanus salvator*, (c) a plerocercoid measured approximately 14 cm length, (d) photomicrograph of anterior end of plerocercoid stage of *Spirometra* parasite using carmine staining method and 100× magnification.

The presence of plerocercoid (spargana) in water monitor lizards was examined according to the methods described by Ooi *et al*. [22]. The water monitor lizards were euthanized, using ethyl ether anesthesia, to be weighed and skinned (Figure-1a). The muscles and subcutaneous tissues were cautiously observed macroscopically for the presence of plerocercoids. The plerocercoids were then removed from the muscles or subcutaneous tissues and placed in a Petri dish containing physiological saline to observe their movement. The number of plerocercoids collected from each infected water monitor lizard was cautiously counted to estimate the intensity of sparganosis infection. The wet preparation was done using carmine staining and clearing with glycerin and then examined using a light microscope with 40× and 100×, and plerocercoids were identified as the larval infective stadium.

## Results

Sparganosis occurrence was recorded from 313 water monitor lizards, and the total prevalence was 69.64% in the total examined samples during the present study. The prevalence of sparganosis in wild-caught and captive-bred water monitor lizards was dominated by adult age groups and recorded at 93.3% and 45.4%, respectively (Table-1). A total of 393 plerocercoids (spargana) were successfully collected from the muscles and subcutaneous tissues during the study (Figure-1b). More than half of the plerocercoids, as the infective stage of *Spirometra* parasites, were located in the muscular tissue of the water monitor lizards, with a total of 280 (71.24%) plerocercoids. Meanwhile, 113 (28.75%) plerocercoids were located in the subcutaneous tissues (Table-1). The prevalence and infection intensities of sparganosis differed according to the water monitor lizard age and source of samples. Wild-caught water monitor lizards had higher prevalence and infection intensities compared to captive breed water monitor lizards. Moreover, wild-caught water monitor lizards in the adult age group had the highest prevalence (93.3%) among the other age groups. A prevalence rate of 45.9% and 79% was recorded in the hatchling and juvenile age groups, respectively. Further, a lower prevalence rate (45.4%) was recorded for the captive-bred water monitor lizards in the adult age group, compared to wild-caught samples. The hatchling and juvenile age groups had 33.3% and 40% prevalence rates, respectively (Table-1). In addition, plerocercoids were macroscopically identified as flat, thin, and white in color with a ribbon-like structure. Furthermore, plerocercoid frequently found in groups in almost all parts of muscular and subcutaneous tissues of water monitor lizard samples. All of the plerocercoid stadiums collected during the study had an average length of 14 cm and average width of 0.4 cm (Figure-1c). Microscopic examination using the carmine staining method showed that the plerocercoids had a segmented body and a mouth-like shape at the top of the anterior side (Figure-1d). Therefore, the results of microscopic observation confirmed that the plerocercoid collected in the present study was the infective stage of *Spirometra* tapeworm parasite or spargana.

**Table-1:** Essential information regarding source, prevalence, intensity, and locations of plerocercoid infection of the 313 wild‑caught and captive‑bred monitor lizard samples from reptile markets in East Java Province, Indonesia.

*Varanus salvator*	Source of samples	Number of samples	Prevalence (%)	Intensity of infection	Number of plerocercoid in subcutaneous tissues	Number of plerocercoid in muscles
Wild-caught						
Hatchling (1-5 months)	Mojokerto	37	17/45.9	0-8	3	16
Juvenile (6-9 months)	Sidoarjo	86	68/79	0-43	29	54
Adult (>10 months)	Banyuwangi	105	98/93.3	0-78	61	127
Captive-bred						
Hatchling (1-5 months)	Gresik	12	4/33.3	0-2	0	6
Juvenile (6-9 months)	Surabaya	40	16/40	0-9	8	29
Adult (>10 months)	Mojokerto	33	15/45.4	0-14	12	48
Total of samples		313		0-25.6	113	280
Total prevalence			69.64			

## Discussion

Due to the high prevalence of sparganosis occurrence, reptile consumption poses a great risk for human sparganosis as reptiles and humans act as intermediate hosts of spargana and potentially contribute to disease transmission. A previous study in several Asian countries reported that snakes play an important role in human sparganosis. In Korea, approximately 50% of all cases involved consumption of raw snakes [3]. In Thailand, sparganosis cases reported from all around the regencies with the highest prevalence rate were recorded in the northeastern part of the country, where there is a tradition of eating food prepared from raw or semi-cooked frogs or snake meat [4]. Moreover, the majority of wild-caught snakes in food markets in China contain spargana, which also spreads to different tissues such as subcutaneous, coelom, and snake meat [23]. Spargana infection has also been reported in two captive viper snakes from India, with spargana in the subcutaneous tissues observed during a gross examination [24].

Various body parts or oil extracts have been utilized in the treatment of various diseases throughout Asia. Varanid gallbladders are believed to cure cardiovascular problems, impotency, and liver failure. Thus, gallbladders from *V. salvator* occupy national records in the traditional Asian medicine trade [25]. The fat and oil of *V. bengalensis* have been utilize by native tribes in Pakistan as a remedy for skin infections and have been used to relieve rheumatic pain [26]. In India, *V. bengalensis* meat was believed to strengthen lung muscles in recovering from a lack of oxygen, and powdered *V. bengalensis* meat was used in energy tonics for the relief of clinical respiratory signs, such as asthma [27]. Khatiwada and Ghimire in 2009 [28] reported that the meat of *V. flavescens* was consumed for medical purposes in Nepal, where individuals also believe that it can be an effective treatment not only for asthma but also for infectious diseases, such as tuberculosis and leprosy. The meat of water monitor lizard (*V. salvator*), a large-bodied species of monitor lizard found in South and Southeast Asia, is not frequently consumed as the main source of protein or culinary products in Indonesia [21,29]. However, there are a few local ethnic groups that continue to eat *V. salvator* meat, such as the Bataks in North Sumatra, the Dayaks in Kalimantan, and the Minahasa in North Sulawesi, who consider *V. salvator* to be a nutritious culinary item [30]. In addition, the previous study also reported the consumption of *V. salvator* meat in two village areas on the southwest coast of Java, Indonesia, where most people consider it as an effective traditional treatment for common skin problems, such as *Pityriasis versicolor* and eczema [29].

Spargana as infective larvae can be identified in any part of the human body; however, the majority of clinical cases generally involve their migration to subcutaneous tissue. Sparganosis most frequently manifests as a nodule within the subcutaneous tissue and presents clinically with inflammation or allergic symptoms [31]. In contrast, previous case reports in Korea have described the clinical features of human axillary sparganosis as a palpable mass, similar to an enlarged lymph node, without inflammatory reactions, such as fever or painful swelling [32]. Sparganosis transmission in humans can be occurred through contaminated water or food, particularly raw reptile meats [23]. The most common route of disease transmission is through drinking contaminated water containing procercoids larvae, which leads them to the intestine, from where they continue to migrate to the muscle or subcutaneous tissue. The second infective possibility is the ingestion of raw or partially cooked frogs, snakes, fish, or even chickens containing plerocercoid larvae [1,33]. Thus, nodules within subcutaneous tissue occur as the result of plerocercoids or spargana migration. Moreover, other routes of transmission include the poultice applications of frog skin placed on open wounds. In general, the typical route of infection in human cases is the direct consumption of intermediate hosts, such as reptiles or amphibians meat, or ingestion of undercooked pigs or wild boars [3,8]. All of the sample sources in this study were located in the East Java Province, Indonesia, which is a predominantly Muslim area, where *V. salvator* is generally not consumed as a regular culinary item or source of protein due to religious beliefs. Therefore, individuals who had eaten *V. salvator* raw or partially cooked meat generally mention that they seek medical benefits, although it was not clear if this was for religious reasons or simply due to a dislike for *V. salvator* meat, considering that the present study was recorded a higher intensity of spargana infection in meat. The previous studies in Indonesia also recorded sparganosis occurrence in reptiles and amphibians, which have played roles as spargana intermediate hosts, such as the oriental rat snake (*P. mucosus*), Javan spitting cobra (*N. sputatrix*), white-lipped pit viper (*Trimeresurus insularis*), and Asian wild frog (*R. rugulosa*) [16,18,20,34].

The present study in water monitor lizard (*V. salvator*) is the first report of sparganosis occurrence in other species of reptile hosts in Indonesia, with a high prevalence rate. Wild-caught water monitor lizards were recorded to have a higher prevalence rate compared to captive breeds, which may be related to their natural environment and the availability of living prey. Regarding the wildlife food chain, wild-caught water monitor lizards frequently consume their prey, which probably already containing spargana, such as frogs, snakes, or smaller lizards. Otherwise, captive water monitor lizards are generally provided proper health care by their owner, and most owners use healthy living prey from certified breeding farms, such as rats and chickens, to reduce the risk of infection by spargana. Ultimately, it is necessary to increase our awareness and stop culinary habits related to wild animal meat or products. The majority of local restaurants provide reptiles, such as wild-caught snakes or water monitor lizards, as culinary products, increasing the risk of sparganosis. Local governments should support and strengthen the food safety inspections of local restaurants that provide water monitor lizard meat as the main course. Moreover, collaborative education efforts should be undertaken for all restaurant owners to only provide meat from certified captive breeding farms or frozen meat to the customer, or completely stop offering reptiles as culinary products to reduce sparganosis transmission from animals to humans.

## Conclusion

Based on the present study, we conclude that several local restaurants that sell reptiles as culinary products have the potential for sparganosis transmission. Although the overall number of restaurants in Indonesia is smaller than in other Asian countries, it still becomes a risk factor that may result in human sparganosis cases. Epidemiological databases, such as those recording the prevalence rate, should be much more enriched with correctly identified spargana for the precise diagnosis not only in reptiles as intermediate hosts but also in humans. Moreover, regarding wild-caught reptiles playing the role of the most potential risk factor, local government laws should make efforts to effectively control wildlife trade, particularly in traditional markets, and also establish collaboration programs with local communities to increase public awareness regarding sparganosis as a neglected zoonotic disease that can have a great impact in Indonesia.

## Authors’ Contributions

AY: Supervised the study, project leader, data analysis, and supported in sample collection. RNP: Carried out examination and dissection of samples. AMK: Carried out the identification and collection of water monitor lizard samples. All authors contributed to the drafting and revision of the manuscript. All authors read and approved the final manuscript.
